# Incidence and risk factors of spinal cord stimulation for persistent or recurrent pain after lumbar spine surgery: a population-based study

**DOI:** 10.1007/s00701-022-05268-w

**Published:** 2022-06-17

**Authors:** Jukka Huttunen, Mikael von und zu Fraunberg, Tiina-Mari Ikäheimo, Henna-Kaisa Jyrkkänen, Mette Nissen, Ville Leinonen, Jyrki Salmenkivi, Antti Malmivaara, Joonas Sirola, Reijo Sund

**Affiliations:** 1 Institute of Clinical Medicine, University of Eastern Finland, Kuopio, Finland; 2 Department of Neurosurgery, Kuopio University Hospital, Kuopio, Finland; 3 Neurosurgery of KUH NeuroCenter, Kuopio University Hospital, PB 100, 70029 KYS Kuopio, Finland; 4 Department of Neurosurgery, Oulu University Hospital, Oulu, Finland; 5 Department of Orthopedics, Helsinki University Hospital, Helsinki, Finland; 6 Health Economics and Equity in Health Care Unit, Finnish Institute for Health and Welfare, Helsinki, Finland; 7 Department of Orthopaedics, and Traumatology and Hand Surgery, Kuopio University Hospital, Kuopio, Finland

**Keywords:** Spinal cord stimulation, Persistent spinal pain syndrome, Lumbar disk herniation, Spinal stenosis, Degenerative disk disease, Spondylolysis and spondylolisthesis

## Abstract

**Purpose:**

This study aims to elucidate the incidence of and independent risk factors for spinal cord stimulator implantations for patients who underwent lumbar spine surgery.

**Methods:**

The PERFormance, Effectiveness, and Cost of Treatment (PERFECT) episodes database, which was established for selected diseases and procedures in Finland, includes all patients who underwent lumbar spine surgery for degenerative spine conditions or spinal cord stimulation (SCS) in Finland from 1986 to 2018. The data on age, sex, hospital diagnoses, surgical procedures, and causes of death were imported from the Finnish national registers into the PERFECT database.

**Results:**

Between 1986 and 2018, 157,824 patients had their first lumbar spine procedure and for 1769 (1.1%) of them, a subsequent SCS procedure was observed during the follow-up. The cumulative incidence of SCS for persistent or recurrent pain after lumbar disk herniation, spinal stenosis, degenerative disk disease, and spondylolysis and spondylolisthesis surgery at 15 years was 1.2%, 1.0%, 2.7%, and 2.6% respectively. At 15 years, the cumulative incidence of SCS for persistent or recurrent pain after lumbar spine surgery after five or more lumbar spinal operations was 11.9%.

**Conclusion:**

Repeated surgery was the most prominent significant risk factor for SCS for persistent or recurrent pain after lumbar spine surgery. The risk of SCS for persistent or recurrent pain after lumbar spine surgery increases significantly along with the number of lumbar spine procedures. When considering repeated lumbar spine surgery, careful evaluation of treatment options should take place to ensure good patient outcomes.

## Introduction

Persistent or recurrent pain after lumbar spine surgery denotes a condition, where despite careful diagnosis and a successful operation, the patient may still experience pain after spinal surgery [2]. Persistent spinal pain syndrome (PSPS) has replaced the previous inadequate term failed back surgery syndrome (FBSS) [2]. In most cases, before persistent or recurrent spinal pain following spinal surgery, repeated lumbar spine interventions have been tried unsuccessfully [8, 9]. The term FBSS is misleading, however, as it does not necessarily have any association with the initial spine surgery [7, 16]. In the treatment of persistent or recurrent spinal pain following spinal surgery, pain relief may be unsatisfactory with drugs and rehabilitation alone [1, 11].

In spinal cord stimulation (SCS), therapeutic doses of electrical current are delivered from the epidural space to spinal cord structures like the dorsal column. SCS induces paresthesia, which decreases discomfort and pain in the affected area, but the actual mechanisms of pain relief in SCS are still unclear. If the patient does not respond to conventional treatment, SCS is a commonly used and good option in persistent or recurrent spinal pain following spinal surgery, effectively reducing pain [5, 6, 8].

The incidence and risk factors of persistent or recurrent spinal pain following spinal surgery and SCS after lumbosacral surgery remain unclear. Previous studies have quoted failure rates after spinal surgery of between 10 and 40% [14]. Not all patients with persistent or recurrent spinal pain following spinal surgery receive SCS devices; this study elucidates current trends in the treatment of persistent or recurrent spinal pain following spinal surgery.

The PERFormance, Effectiveness, and Cost of Treatment (PERFECT) project was established to monitor the content, quality, and cost-effectiveness of treatment episodes in medical care in Finland, including surgical procedures involving the lumbar spine [3, 4, 12]. In this study, we analyzed the cumulative incidence of and independent risk factors for spinal cord stimulator implantations for persistent or recurrent pain after lumbar spine surgery between 1986 and 2018 in a nationwide population-based study.

## Materials and methods

### Finnish PERFormance, Effectiveness, and Cost of Treatment back database

The PERFECT research database, which was established for selected diseases and procedures with significance in terms of costs and patient numbers, includes all patients in Finland who have undergone lumbar spine surgery for degenerative spine conditions. The database was created by the Finnish Institute for Health and Welfare (THL), an independent agency under the supervision of the Finnish Ministry of Social Affairs and Health [3, 4, 12].

The PERFECT database utilized the THL-maintained Finnish Hospital Discharge Register (FHDR) and the Care Register for Health Care (CRHC) to identify all lumbar spine operations and spinal cord stimulator implantations in Finland from 1986 to 2018. The data on age, sex, hospital diagnoses, surgical procedures, and causes of death were imported from the Finnish national registers into the PERFECT database.

The quality of the FHDR and CRHC data has been shown to be excellent: the completeness of the identification of hospitalized persons within recent years is over 95%. The accuracy of the diagnosis has been studied, and the positive predictive value was between 75 and 99%. For rare diseases, the likelihood of false positives was higher [13].

The PERFECT research database was approved by the Ethics Committee of the THL (THL 496/6.02.00/2011), and the respective authorities of the administrative registers approved the combining of the data. Researchers had access only to the anonymized data. We did not contact the patients during the study, and therefore informed consent was not required from the patients.

### Study population

We identified patients from the FHDR and CRHC who underwent lumbar spinal procedures between 1986 and 2018 using the specific surgical procedure operational codes from the Finnish version of the Nordic Medico-Statistical Committee classification (used since 1996) and the Finnish Hospital League (FHL) classification (for the years 1986–1996). The operational codes retrieved included those for lumbar spine procedures: ABC07, ABC16, ABC26, ABC36, ABC56, ABC66, ABC99, NAG60-67, NAG99, and NAB92 (FHL codes 9211–9219 and 9181–9189). The operational codes for SCS included ABD30 and ABD32 (and 2324).

We also retrieved all the recorded diagnostic codes between 1986 and 2018 for lumbar spinal procedures. The ICD-8 (1969–1986), ICD-9 (1987–1995), and ICD-10 (1996-) codes retrieved included diagnoses for herniated intervertebral disk: M51.1 (ICD-10), 7221A, 7227C, 7228C (ICD-9), 35,399, 72,510, 72,519, 72,599, and 72,880 (ICD-8) and G55.1; spinal stenosis: M47.1, M47.2, M47.9, and M48.0 (ICD-10), 7213A, 7214A, 7218X, 7219X, 7240B, 7244A (ICD-9); degenerative disk disease: M47.82 and M51.3 (ICD-10), 7225A, and 7225B (ICD-9); and spondylolysis and spondylolisthesis: M43.0 and M43.1 (ICD-10), 7385A, 7561A, 7561D (ICD-9). We classified the patients into five groups according to specific lumbar spine operation and diagnosis: disk herniation, spinal stenosis, degenerative disk disease, spondylolysis and spondylolisthesis, and other lumbar spine procedures.

The inclusion criteria for this study cohort were adult patients ≥ 18 years and patients with their first observed lumbar spine operation after 1986 (index surgery).

We identified all SCS patients from the patient cohort of lumbar spine procedures with a diagnosis of herniated intervertebral disk, spinal stenosis, degenerative disk disease, and spondylolysis and spondylolisthesis and according to the operational codes for SCS. If the patient had an SCS operational code after the lumbar spine operation, the patient was considered to have had SCS therapy for persistent or recurrent pain after lumbar spine surgery. The patient’s age and gender were identified at the first lumbar spine operation and at the SCS procedure. The patients were followed up for the SCS procedure from the first lumbar spine operation until the end of 2018 or death.

### Statistical analysis

The discrete variables were expressed in proportions, and the continuous variables were presented as means. We calculated the cumulative incidence of spinal cord stimulator implantations after lumbar spine surgery with the competing risk analysis method. The independent risk factors for SCS were analyzed using Cox proportional hazards regression. The covariates in the cause-specific Cox regression analysis were sex, age, type of lumbar spine procedure, and number of lumbar spine operations. In addition, year of the lumbar spine procedure was used as strata in the Cox model. A test for proportional hazards assumption indicated potential violation of the assumption, but as visual inspections did not indicate any large problems and as the estimated Fine-Gray model yielded almost the same estimates as the Cox model, we report the results from the Cox model.

## Results

### Study population

Altogether, 157,824 patients had 198,158 lumbar spinal operations between 1986 and 2018 in Finland. Out of 198,158 operations, 105,370 were for lumbar disk herniation, 74,572 were for lumbar spinal stenosis, 10,644 were for degenerative disk disease in the lumbar spine, and 7572 were for spondylolysis and spondylolisthesis. Table 1 presents the yearly lumbar spine operations in Finland between 1986 and 2018.

**Table 1:** Lumbar spine procedures for degenerative spine conditions in Finland between 1986 and 2018 according to the Finnish Institute for Health and Welfare (THL)

Year	Disk herniation	Spinal stenosis	Degenerative disk disease	Spondylolysis and spondylolisthesis
1986	1972	538	108	2
1987	2589	672	68	96
1988	2411	778	64	128
1989	2676	704	73	124
1990	2875	727	77	110
1991	2973	769	101	98
1992	3257	957	103	120
1993	3699	1126	140	121
1994	4288	1345	162	130
1995	4083	1362	175	116
1996	4029	1223	180	142
1997	4090	1478	189	187
1998	4038	1639	272	191
1999	3985	1738	297	187
2000	3840	1802	304	203
2001	3417	1624	284	195
2002	3520	1852	311	210
2003	3289	2058	343	252
2004	3135	2287	437	233
2005	3027	5820	420	262
2006	3056	2548	435	267
2007	3074	2672	421	256
2008	2984	2802	577	315
2009	2944	2947	496	263
2010	2969	3030	488	291
2011	2869	3169	473	300
2012	2920	3375	529	319
2013	2894	3473	552	334
2014	2879	3742	636	374
2015	2966	3946	529	418
2016	2836	4126	466	436
2017	2787	3946	439	429
2018	2999	4297	495	463

Out of the 19,840 patients who died between 1986 and 2018, 7598 underwent a procedure for lumbar disk herniation, 11,346 had a procedure for lumbar spinal stenosis, 535 had a procedure for degenerative disk disease in the lumbar spine, and 361 had a procedure for spondylolysis and spondylolisthesis.

### Spinal cord stimulation for persistent or recurrent pain after lumbar spine surgery

Altogether, 4944 patients had their first spinal cord stimulator implanted between 1986 and 2018. Out of these, 1769 (36%) had their SCS device implanted because of persistent or recurrent pain after lumbar spine surgery. The mean age of the persistent or recurrent pain after lumbar spine surgery patients who underwent an SCS procedure was 54 years at 2018 and proportion of men was 46%. Table 2 presents the yearly variations in SCS operations in Finland between 1986 and 2018.

**Table 2:** Spinal cord stimulator (SCS) implantations for chronic pain after lumbar spine procedures in Finland between 1986 and 2018 according to the Finnish Institute for Health and Welfare (THL)

Year	All SCS implantations in Finland	SCS implantations for chronic pain after lumbar spine procedures (% of all SCS)	Age of patients with chronic pain after lumbar spine procedures and SCS (mean)	Proportion of male gender from SCS implantations for chronic pain after lumbar spine procedures
1986–1989	31	0 (0%)		
1990–1992	33	5 (15%)	43	80%
1993–1995	92	19 (21%)	50	63%
1996	78	27 (35%)	50	63%
1997	82	28 (34%)	48	46%
1998	73	31 (42%)	43	68%
1999	63	25 (40%)	49	48%
2000	85	39 (46%)	45	64%
2001	65	29 (45%)	49	72%
2002	100	36 (36%)	46	50%
2003	139	59 (42%)	47	63%
2004	156	61 (39%)	48	51%
2005	160	63 (39%)	47	46%
2006	129	40 (31%)	48	30%
2007	107	43 (40%)	46	44%
2008	169	61 (36%)	52	41%
2009	186	64 (34%)	48	48%
2010	217	81 (37%)	51	52%
2011	308	106 (34%)	51	45%
2012	362	115 (32%)	52	50%
2013	342	110 (32%)	50	50%
2014	369	148 (40%)	51	49%
2015	398	133 (33%)	54	43%
2016	421	168 (40%)	53	48%
2017	385	132 (34%)	53	48%
2018	394	146 (37%)	54	46%

### Annual spinal cord stimulation rates for persistent or recurrent pain after lumbar spine surgery

The first SCS device because of persistent or recurrent pain after lumbar spine surgery was implanted in Finland in 1990, after which the number of yearly implantations increased. There were 39 SCS device implantations in 2000 and 81 in 2010. At the end of the study period, in 2018, there were 146 SCS device implantations. Between 2000 and 2018, there was a 270% increase in SCS device implantations for persistent or recurrent pain after lumbar spine surgery, and the proportion of all SCS implantations in Finland with a diagnosis of persistent or recurrent pain after lumbar spine surgery has remained stable at between 31 and 46% (Table 2).

### Cumulative incidence of spinal cord stimulation for persistent or recurrent pain after lumbar spine surgery

**Fig. 1:**
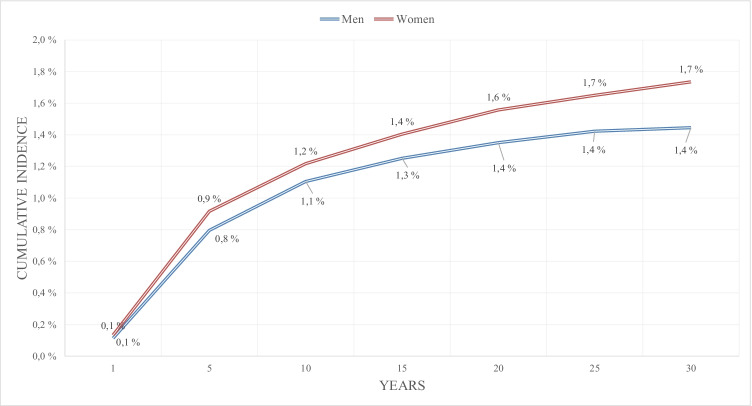
Cumulative incidence of spinal cord stimulation for failed back surgery syndrome after first lumbar spine operation according to the gender in 157,824 patients with 198,158 lumbar spinal operations between 1986 and 2018 in Finland

**Fig. 2:**
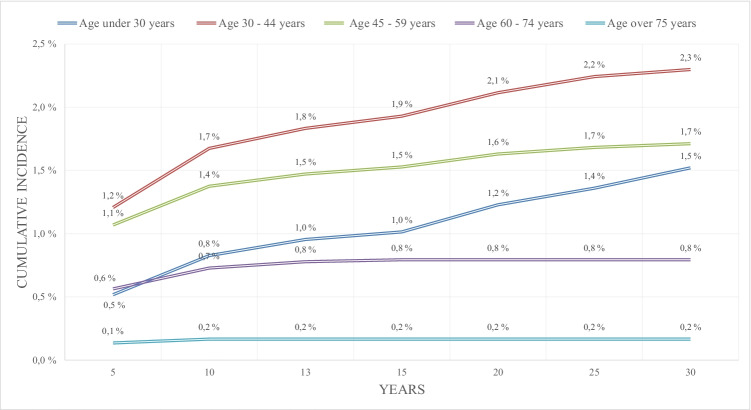
Cumulative incidence of spinal cord stimulation for failed back surgery syndrome after first lumbar spine operation according to the age in 157,824 patients with 198,158 lumbar spinal operations between 1986 and 2018 in Finland

**Fig. 3:**
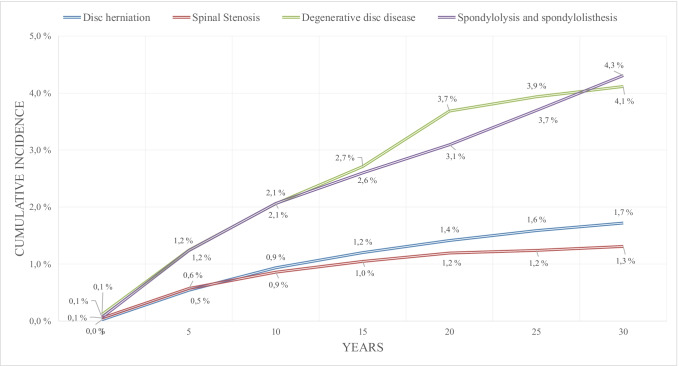
Cumulative incidence of spinal cord stimulation for failed back surgery syndrome after first lumbar spine operation according to specific lumbar spine diagnoses in 157,824 patients with 198,158 lumbar spinal operations between 1986 and 2018 in Finland

The cumulative incidence of SCS for persistent or recurrent pain after lumbar spine surgery for women at 15 years was 1.4% (confidence interval [CI] 1.31–1.50%) and for men 1.3% (CI 1.17–1.34%) at 15 years (Fig. 1). Figure 2 presents the cumulative incidence of SCS for persistent or recurrent pain after lumbar spine surgery according to age of the patient. Age group 30 to 44 years had the highest cumulative incidence of 1.9% (CI 1.79–2.07%) at 15 years for SCS for persistent or recurrent pain after lumbar spine surgery (Fig. 2). Of the 157,824 patients included in the 30-year cumulative incidence analysis, 86,954 had lumbar disk herniation procedures, 58,376 had lumbar spinal stenosis procedures, 6167 had degenerative disk disease procedures in the lumbar spine, and 6327 had spondylolysis and spondylolisthesis as their first procedures in the lumbar spine. The cumulative incidence of SCS for persistent or recurrent pain after lumbar spine surgery for disk herniation, lumbar spinal stenosis, degenerative disk disease in the lumbar spine, and spondylolysis and spondylolisthesis at 5 years was 0.5% (CI 0.49–0.59%), 0.6% (CI 0.50–0.64%), 1.2% (CI 0.95–1.54%), and 1.2% (CI 0.94–1.53%), respectively, and at 15 years 1.2% (CI 1.12–1.28%), 1.0% (CI 0.94–1.15%), 2.7% (CI 2.21–3.22%), and 2.6% (CI 2.11–3.09%), respectively (Fig. 3).

**Fig. 4:**
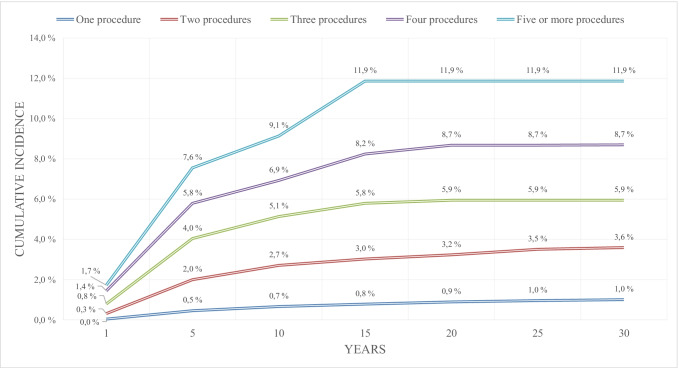
Cumulative incidence of spinal cord stimulation for failed back surgery syndrome after first lumbar spine operation according to the number of the patients last recorded lumbar spine operation in 157,824 patients with 198,158 lumbar spinal operations between 1986 and 2018 in Finland

Figure 4 presents the cumulative incidence of SCS for persistent or recurrent pain after lumbar spine surgery according to the number of observed lumbar spinal operations. At 5 years, the cumulative incidence of SCS for persistent or recurrent pain after lumbar spine surgery was 7.6% (CI 4.88–10.24%) after five or more lumbar spinal operations and at 15 years, the cumulative incidence of SCS for persistent or recurrent pain after lumbar spine surgery after five or more lumbar spinal operations was 11.9% (CI 7.53–16.21%). Patients with only one observed lumbar spine operation had the lowest incidence of SCS for persistent or recurrent pain after lumbar spine surgery at 15 years 0.8% (CI 0.73–0.85%) (Fig. 4).

### Independent risk factors for spinal cord stimulation for persistent or recurrent pain after lumbar spine surgery

In the Cox regression analysis, female sex (hazard ratio [HR] 1.22); age groups 30–44 years (HR 1.42), 60–74 (HR 0.40), and over 74 (HR 0.09) in comparison to age group less than 30 years; spinal stenosis (HR 1.72), degenerative disk disease (HR 2.69), and spondylolisthesis (HR 2.53) in comparison to disk herniation; and two lumbar spine operations (HR 3.43), three lumbar spine operations (HR 5.58), four lumbar spine operations (HR 7.02), and five or more lumbar spine operations (HR 9.66) in comparison to only one observed lumbar spine operation were risk factors for SCS for persistent or recurrent pain after lumbar spine surgery after adjustments for these factors and the year of operation (Table 3).

**Table 3:** Independent risk factors for spinal cord stimulation (SCS) for chronic pain after lumbar spine surgery using Cox regression analysis in 157,824 patients with 198,158 lumbar spinal operations in Finland between 1986 and 2018

Variable		*n*	Cumulative incidence of SCS at 30 years	HR	*p* value	CI (95%)
Sex	Men	81,832	1.4%	ref		
	Women	75,992	1.7%	1.22	*p* < 0.001	1.11–1.34
Age	< 30	12,560	1.5%	ref		
	30–44	42,284	2.3%	1.42	*p* < 0.001	1.42–2.10
	45–59	47,721	1.7%	1.00	*p* = 0.977	0.82–1.23
	60–74	38,544	0.8%	0.40	*p* < 0.001	0.32–0.51
	75 >	16,715	0.2%	0.09	*p* < 0.001	0.06–0.14
Type_of_lumbar spine procedure	Disk herniation	81,126	1.7%	ref		
	Spinal stenosis	60,814	1.3%	1.72	*p* < 0.001	1.52–1.94
	Degenerative disk disease	8486	4.1%	2.69	*p* < 0.001	2.33–3.11
	Spondylolysis and spondylolisthesis	6398	4.3%	2.53	*p* < 0.001	2.10–3.05
Number of_lumbar spine operations	1	127,571	1.0%	ref		
	2	23,189	3.6%	3.43	*p* < 0.001	3.08–3.81
	3	5304	5.9%	5.58	*p* < 0.001	4.80–6.48
	4	1265	8.7%	7.02	*p* < 0.001	5.49–8.98
	5 or more	495	11.9%	9.66	*p* < 0.001	6.85–13.63

## Discussion

In the present study, we analyzed comprehensive information on lumbar spinal surgeries in Finland between 1986 and 2018, and this study had complete follow-up on all SCS implantations in Finland during the study period for the analysis of risk factors for SCS for persistent or recurrent pain after lumbar spine surgery. These surgeries in Finland increased 270% between 2000 and 2018, most likely because SCS therapy has become a widely accepted therapy for chronic pain and has had good results in the treatment of persistent or recurrent spinal pain following spinal surgery [1, 9, 11].

The risk of SCS for persistent or recurrent pain after lumbar spine surgery after only one lumbar spine procedure is very low 0.8% at 15 years and 1.0% at 30 years. The risk of SCS for persistent or recurrent pain after lumbar spine surgery is notably associated with the increasing number of lumbar spine operations. At 15 years, the cumulative incidence of SCS for persistent or recurrent pain after lumbar spine surgery after 5 or more lumbar spinal operations was 11.9%. Repeated lumbar spine surgery is not necessarily the solution for persistent or recurrent spinal pain following spinal surgery, especially if neuropathic pain is involved without lumbar spine instability [10, 15]. Repeated lumbar spine surgery might have a negative effect on the long-term outcome. Secondary or repeated surgery is less likely to have a good outcome compared to the primary surgery for persistent or recurrent spinal pain following spinal surgery without lumbar spine instability [10, 15]. SCS has had good results compared to repeated surgery and should be considered an option when the outcome of repeated lumbar spine surgery is predicted or known to be poor [5, 6, 8, 10].

During the period under study, the annual number of lumbar spine procedures for degenerative disk disease and spondylolysis and spondylolisthesis increased. Patients with a diagnosis of spondylolysis and spondylolisthesis or degenerative disk disease in the lumbar spine for their lumbar spinal procedure are also at increased risk for SCS for persistent or recurrent pain after lumbar spine surgery compared to patients with diagnoses of spinal stenosis or lumbar disk herniation for their procedures. Degenerative disk disease, spondylolysis, and spondylolisthesis may cause more persistent or recurrent pain after lumbar spine surgery and SCS, because the underlying degenerative process in these diagnoses causes longer-term disability, and symptoms may be more progressive than disk herniation, which has more rapid symptoms and a tendency to spontaneously heal. Unfortunately, we do not have specific information on the selected treatment on the basis of the diagnosis before lumbar surgery, but degenerative disk disease and spondylolysis and spondylolisthesis may have required more extensive surgery with fusion of lumbar segments and thus caused the higher incidence of SCS for persistent or recurrent pain after lumbar spine surgery.

In our study, younger age was associated with increased risk, and older age was associated with decreased risk of SCS for persistent or recurrent pain after lumbar spine surgery. Possibly, younger patients are more easily considered to have persistent or recurrent spinal pain following spinal surgery and neuropathic pain dominance in the presence of anatomically normal post-surgical findings, but possibly patients under 30 are not prepared to have just pain alleviating SCS device implantations but would prefer corrective lumbar spine surgery instead [10, 15]. Degenerative changes in the lumbar spine are more common in older patients; hence, it is possible that persistent or recurrent spinal pain following spinal surgery is more likely to be labeled as such with nociceptive pain dominance, explaining the increased risk in younger patients and decreased risk in older patients for SCS and persistent or recurrent spinal pain following spinal surgery. Management of chronic pain may be more complex in older patients due to comorbidities; also, older patients maybe more willing to accept disability compared to younger patients and decreased risk in older patients for SCS and hence, it is possible that persistent or recurrent spinal pain following spinal surgery is more likely to after lumbar spine surgery. Persistent or recurrent spinal pain following spinal surgery is a common term for chronic and intractable pain after lumbar spine surgery. In the present study, all patients have diagnosis indicating lumbar spine pathology before lumbar spine surgery and have had at least one lumbar spine procedure before their SCS procedure. Because of the retrospective register study design, some of the patients labeled as SCS for persistent or recurrent pain after lumbar spine surgery may possibly have had the SCS device with a diagnosis not related to lumbar spine surgery. In this register-based retrospective study, reliant on the PERFECT research database and operational coding, we did not have information on patient-reported outcome. In the present study, the incidence of SCS for persistent or recurrent pain after lumbar spine surgery was very low in the patients with only one lumbar spine procedure and increased along with the number of lumbar spine procedures confirming the relation between SCS and lumbar spine surgery indicating that the etiology for SCS procedure was recurrent pain after lumbar spine surgery. Proportion of SCS patients may have had additional lumbar spine surgery after SCS device implantation, but the present study was not designed to address lumbar spine procedures after SCS device implantations.

The Finnish healthcare system is almost free of charge to the patients and taxpayer-funded, which most likely decreases differences that possibly derive from socioeconomic diversity and allows access to healthcare for everyone. The Finnish healthcare system covers SCS and lumbar spine operations, and, in the present study, there was no or minimal selection because of the patient’s economic situation. Confirmed lumbar spine diagnoses and the lumbar spine procedures of the patients are reliable due to the FHDR and CRHC data [13]. Accurate data from the Finnish registers allowed the long follow-up time and ensured that patients were not lost during the follow-up, but it is possible that patients with lumbar spine surgery have emigrated from Finland during the study period.

## Conclusions

SCS therapy has become a common therapy in the treatment of persistent or recurrent pain after lumbar spine surgery. Patients’ risk of SCS for persistent or recurrent pain after lumbar spine surgery increases significantly with the number of lumbar spine procedures. When considering repeated lumbar spine surgery, careful evaluation of treatment options between spinal cord stimulation and lumbar spine surgery should take place to ensure good patient outcomes.

## Declarations

The manuscript submitted does not contain information about medical device(s)/drug(s).
